# Arthritis Mutilans: A Devastating Manifestation of Psoriatic Disease Without Dactylitis in a 61-Year-Old Patient

**DOI:** 10.7759/cureus.88502

**Published:** 2025-07-22

**Authors:** Azalea Guadalupe Altamirano De La Cruz, Francisco Javier Lugo Rincón-Gallardo, Maria Daniela Salazar López, Celtzin Jazbeth Ruedas Rodriguez, Jennifer Miriam Mendoza Gómez

**Affiliations:** 1 Internal Medicine, Mexican Social Security Institute (IMSS) General Zone Hospital No. 8, Ensenada, MEX; 2 Internal Medicine, Institute for Social Security and Services for State Workers, Ensenada, MEX; 3 Internal Medicine, General Hospital of the Institute for Social Security and Services for State Workers, Queretaro, MEX; 4 Medical Sciences, University of Guanajuato, Guanajuato, MEX; 5 Internal Medicine, Clinica Hospital B Chetumal, Chetumal, MEX

**Keywords:** arthritis mutilans, bone resorption, dactylitis, joint destruction psoriasis, telescoping fingers

## Abstract

Arthritis mutilans (AM) is the most severe and least common form of psoriatic arthritis (PsA), characterized by digital shortening due to osteolysis and often associated with profound functional disability. We report a case of a 61-year-old male with longstanding plaque psoriasis and poor treatment adherence who developed AM affecting the interphalangeal joints of the left foot, with significant joint deformity and extensive skin involvement (30% body surface area). Imaging revealed severe bone breakdown, leading to the affected phalanges of the foot appearing shortened or telescoped.

AM typically presents in patients with distal interphalangeal joint involvement and is often linked to a history of dactylitis. Although rare, it can also appear in other rheumatic conditions, including rheumatoid arthritis and systemic lupus erythematosus. Current challenges include the absence of standardized phenotypic definitions and a limited understanding of clinical predictors. Early identification of at-risk patients is critical to prevent irreversible joint damage and disability.

## Introduction

Arthritis mutilans (AM) represents the most devastating manifestation of psoriatic arthritis (PsA), characterized by rapid and irreversible joint destruction through aggressive osteolysis (destruction of bone tissue). This destructive process culminates in dramatic digital shortening and the pathognomonic (characteristic and specific) "telescoping deformities," wherein sequential bone collapse creates a characteristic appearance resembling a closed telescope. Such structural devastation invariably results in profound functional impairment, manifesting as severe limitations in essential activities of daily living, including compromised grip strength, ambulatory dysfunction, and impaired self-care capabilities [1-3].

Building upon the foundational work of Moll and Wright, whose seminal 1973 classification established AM as one of the five distinct PsA subtypes [4], contemporary diagnostic frameworks have substantially refined our understanding of this condition. Notably, the 2006 CASPAR criteria (Classification Criteria for Psoriatic Arthritis) have validated AM as an independent predictor of poor long-term prognosis [5], while the most recent International Classification of Diseases, 11th Revision (ICD-11) has formally categorized this entity under "Mutilating Psoriatic Arthritis" (FA21.Y) [6].

Nevertheless, despite these diagnostic advances, AM remains significantly understudied, with current epidemiological data indicating a prevalence of merely 3-5% among PsA patients [7]. Of particular clinical importance is the recognition that AM is not pathognomonic (characteristic and specific) for PsA. Indeed, severe cases demonstrating similar patterns of osteolytic (bone-destroying) destruction have been extensively documented across multiple rheumatologic conditions, including rheumatoid arthritis (particularly seronegative variants) [8], systemic lupus erythematosus (SLE)-associated arthritis [9], systemic sclerosis [10], and juvenile idiopathic arthritis [11]. This considerable similarity in clinical manifestations significantly complicates differential diagnosis and underscores the imperative for precise diagnostic classification.

Although AM has achieved recognition as a well-established clinical entity, recent literature has illuminated several critical unresolved questions that continue to challenge clinical practice.

Firstly, regarding diagnostic conventions, the clinical heterogeneity of AM presentation patterns creates significant diagnostic challenges. While dactylitis (characterized by severe, sausage-like swelling of entire digits) represents a well-established feature in the majority of AM cases [12], the subset of patients developing severe osteolysis through alternative pathways remains inadequately studied. This phenotypic variability fundamentally challenges current diagnostic frameworks and may contribute to delayed recognition of non-classical presentations.

Secondly, concerning predictors in severe cutaneous disease, there exists a paucity of data regarding predictors of rapid AM progression, particularly in patients presenting with extensive cutaneous psoriatic involvement exceeding 30% body surface area (BSA) [13]. This limitation significantly hampers risk stratification and preventive intervention strategies.

Thirdly, with respect to standardized assessment, no currently available radiological scoring system effectively integrates established diagnostic criteria (such as CASPAR) with functional impact metrics to provide comprehensive prognostic guidance and inform optimal management strategies [5,14,15].

These identified gaps collectively contribute to a critical clinical problem: standard disease activity measures and inflammatory markers (including acute phase reactants) may paradoxically appear within normal limits despite the presence of significant joint destruction, thereby leading to systematic underestimation of disease severity and consequent delays in therapeutic intervention [14]. Consequently, these persistent knowledge gaps continue to hinder early identification, accurate prognostic assessment, and optimal management of this devastating condition.

The primary objectives of this case report are to illustrate a characteristic case of mutilans PsA presenting with severe osteolytic (bone-destroying) changes occurring concurrently with normal acute phase reactants, thereby highlighting a key diagnostic challenge in clinical practice, and to examine the inherent limitations of current disease activity measures when applied to destructive PsA subtypes, with particular emphasis on their failure to accurately reflect underlying destructive pathology despite reassuring biomarker profiles.

## Case presentation

A 61-year-old male presented with a severe exacerbation of cutaneous psoriasis and progressive articular deformities in his feet, causing significant functional limitation. His medical history dates back to 2010, when, at age 51, he was diagnosed with localized cutaneous psoriasis affecting approximately 5% of his body surface area on his elbows and knees. Initially, topical corticosteroids provided partial improvement for six months; however, he subsequently developed a loss of therapeutic efficacy.

By 2012, at 53 years old, his cutaneous psoriasis had progressed to encompass 15% of his body surface area, with extension to his trunk and extremities. At this point, methotrexate at 15 mg weekly was initiated, leading to a good initial response over eight months. Nevertheless, the patient self-discontinued the treatment due to poor adherence. In 2013, he developed his initial articular symptoms, characterized by pain in the proximal interphalangeal joints of his hands and morning stiffness. Methotrexate was restarted at 20 mg weekly, but he experienced gastrointestinal intolerance and a limited response, resulting in another discontinuation after four months.

The formal diagnosis of polyarticular psoriatic arthritis was established in 2015, using the CASPAR criteria, where the patient scored four points (current cutaneous psoriasis, documented history of psoriasis, negative rheumatoid factor). At that time, sulfasalazine 2 g daily was initiated but yielded an unsatisfactory response. A critical factor for the case outcome was that the patient completely abandoned systemic treatment for eight months and maintained very poor adherence during the subsequent five years (2015-2020). This prolonged period of therapeutic abandonment directly coincided with established correlations between treatment gaps and disease progression (Table 1).

**Table 1:** Medical history of the patient. BSA: body surface area; PIP: proximal interphalangeal; HAQ-DI: Health Assessment Questionnaire-Disability Index; CASPAR: Classification Criteria for Psoriatic Arthritis; PASI: Psoriasis Area and Severity Index; DMARDs: disease-modifying antirheumatic drugs.

Date	Clinical event	Manifestations	Treatment initiated	Duration	Response	Reason for discontinuation
2010	Initial psoriasis diagnosis	Erythematous plaques on elbows and knees (~5% BSA)	Topical corticosteroids	6 months	Partial improvement	Loss of efficacy/reappearance of plaques in previously controlled areas
2012	Cutaneous progression	Extension to trunk and extremities (~15% BSA)	Methotrexate 15 mg/week	8 months	Good initial response	Poor patient adherence
2013	First articular symptoms	Pain in the PIP joints of the hands, morning stiffness	Methotrexate 20 mg/week	4 months	Limited response	Gastrointestinal intolerance
2015	Psoriatic arthritis diagnosis	Polyarticular arthritis (CASPAR criteria: 4 points)	Sulfasalazine 2 gr/day	8 months	Partial response	Loss of efficacy/severe cutaneous manifestations (PASI >10)
2016-2019	Period without follow-up	Treatment abandonment, disease progression	No systemic treatment	3 years	Significant deterioration	Loss of medical follow-up
2020-2023	COVID-19 pandemic	Progressive worsening, extensive psoriasis (25% BSA)	No systemic treatment	3 years	Severe deterioration	Pandemic restrictions
2024	Current assessment	Arthritis mutilans, telescopic deformities in feet, radiographic osteolysis, severe disability (HAQ-DI: 2.8), extensive psoriasis (30% BSA)	Evaluation for biological therapy	Pending	Pending evaluation	Multiple failed attempts with conventional DMARDs

**Figure 1:**
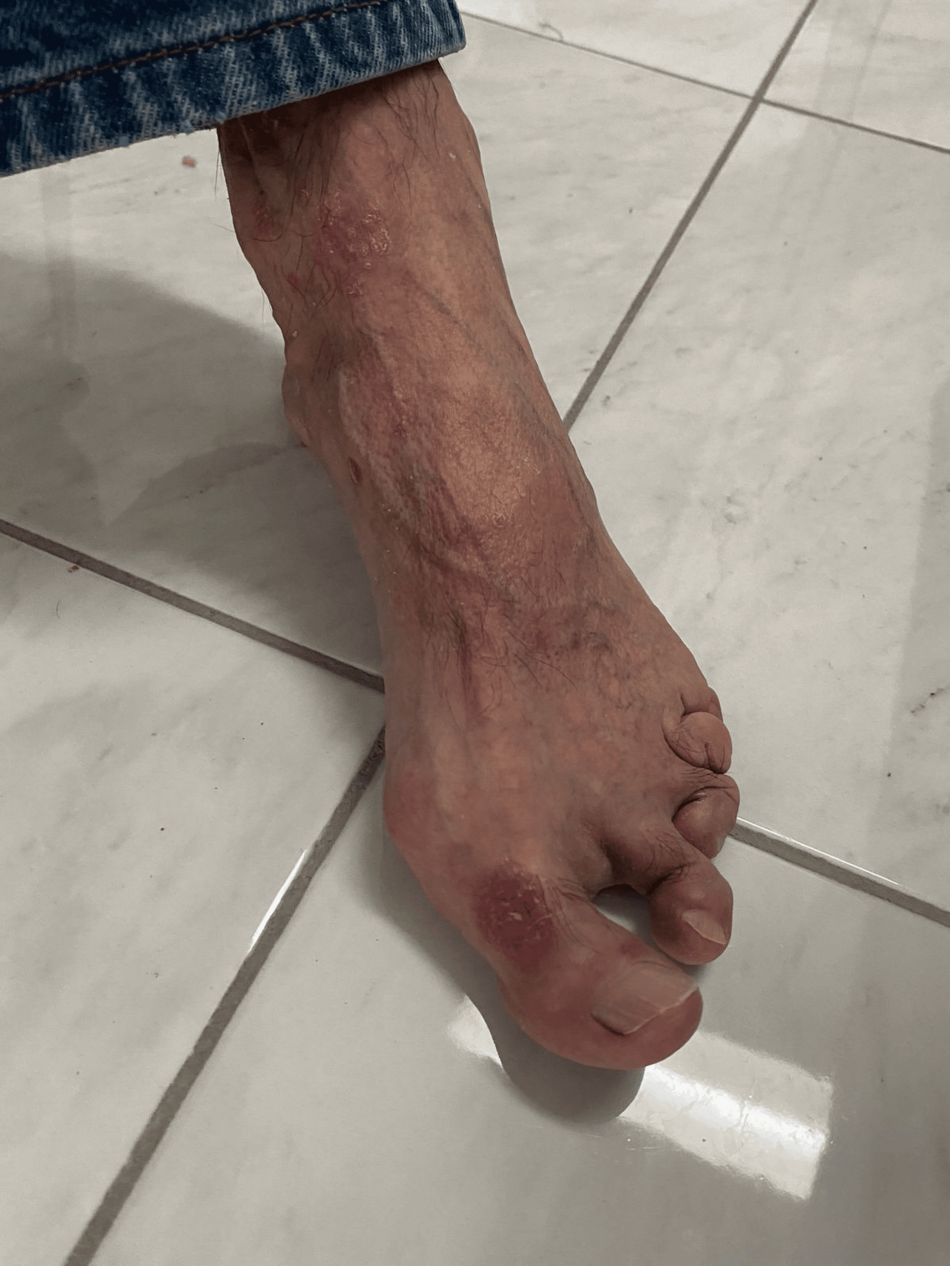
Marked deformities and severe malalignment of the toes, especially the second, third, fourth, and fifth phalanges of the foot.

**Figure 2:**
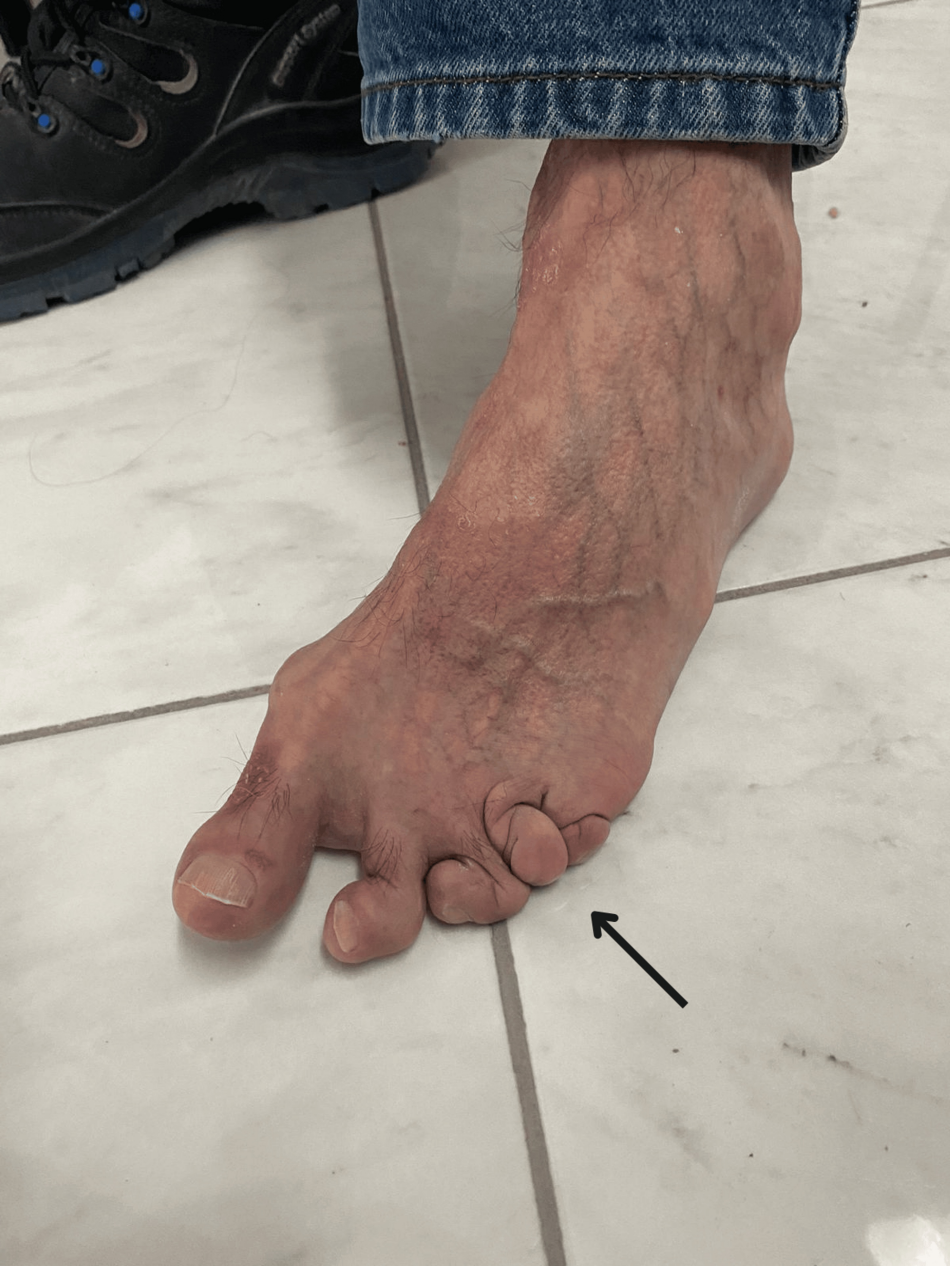
Multiple, well-demarcated, erythematous plaques with varying degrees of silvery scaling are evident on the dorsum of the foot, particularly over the metatarsophalangeal (MTP) joint of the hallux (big toe) and extending onto other and shortening and telescoping appearance of some phalanges of the foot, particularly the third, fourth, and fifth, strongly raise suspicion for arthritis mutilans.

In 2020, at 60 years old, he presented with a severe exacerbation marked by extensive psoriasis (25% body surface area) and active polyarthritis. However, the initiation of biological therapy was unfortunately delayed due to the limitations imposed by the COVID-19 pandemic. By 2021, the patient had developed arthritis mutilans with irreversible telescopic deformities in his phalanges of the foot (Figures 1, 2).

**Figure 3:**
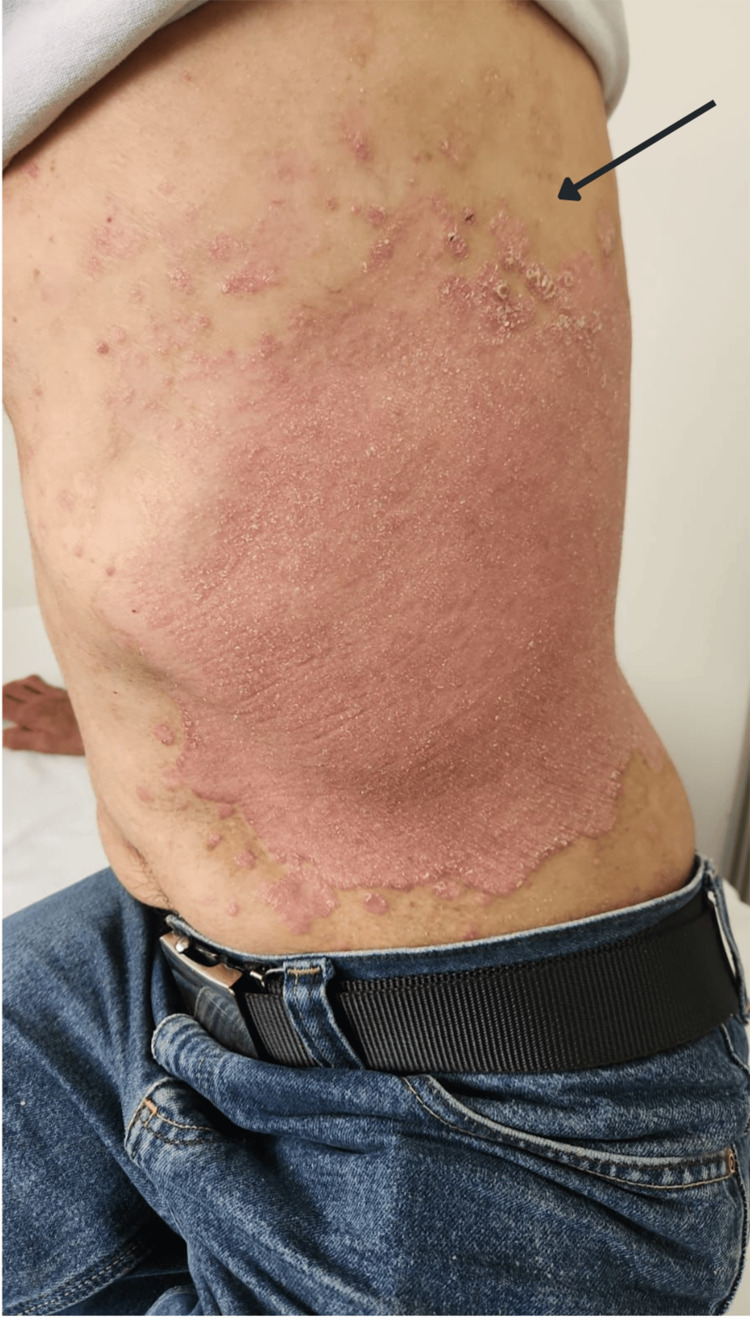
Plaque psoriasis showing well-demarcated, erythematous plaques with overlying silvery-white scales located on the lateral trunk. The arrow indicates a cluster of smaller psoriatic lesions exhibiting the characteristic micaceous scaling. The distribution is consistent with chronic plaque psoriasis.

Anthropometric characteristics and risk factors included a body mass index (BMI) of 28.3 kg/m² (overweight), along with a significant smoking history of 15 pack-years (ceased in 2018). The current physical examination revealed extensive psoriasis with erythematous-squamous plaques, reaching up to 30 cm in diameter on his trunk. These plaques displayed intense underlying erythema covered by adherent silvery scales with clearly defined borders (Figure 3).

**Figure 4:**
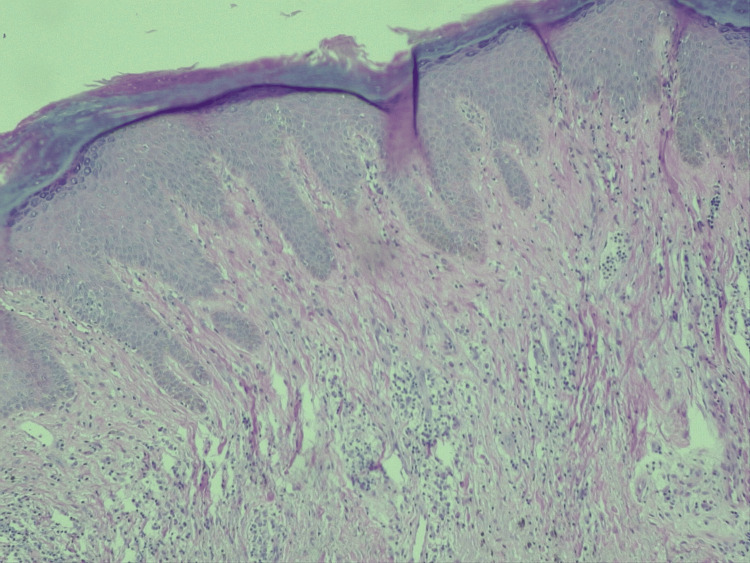
Histological biopsy of psoriatic skin stained with hematoxylin and eosin (H&E) (10x magnification). The image shows characteristic features of psoriasis, including marked epidermal hyperplasia (acanthosis) with regular elongation of rete ridges, diminished or absent granular layer (hypogranulosis), and parakeratosis with retained nuclei in the stratum corneum. Papillomatosis and a perivascular lymphohistiocytic infiltrate in the papillary dermis are also evident.

Auspitz's sign was positive, indicative of characteristic pinpoint bleeding upon scraping. Evaluation using the Psoriasis Area and Severity Index (PASI) score showed a value of 24.1, indicating severe psoriasis affecting 30% of his body surface area. Given the extent and atypical characteristics of the lesions, a skin biopsy was performed, which confirmed parakeratotic hyperkeratosis, thus establishing the definitive histological diagnosis of psoriasis (Figure 4).

Functional evaluation using the Health Assessment Questionnaire-Disability Index (HAQ-DI) revealed a score of 2.8 (range: 0-3), indicating severe functional disability [2]. The patient reported an inability to put on socks and shoes without assistance, severe difficulty standing due to pain and deformity in his feet, the necessity of a cane for walking due to the telescopic deformities, and an inability to stand for prolonged periods. Manual functions remained preserved, with limitations primarily related to the mutilating deformities in his feet [16,17].

Laboratory studies showed negative rheumatoid factor (8 IU/mL) and negative anti-CCP (12 U/mL), ruling out coexisting systemic inflammation and supporting the diagnosis of psoriatic arthritis versus rheumatoid arthritis [8]. C-reactive protein (CRP) was normal (0.2 mg/L), a relevant finding that validated the paradigm that bone destruction in arthritis mutilans can occur without systemic elevation of acute phase reactants (Table 2).

**Table 2:** Initial laboratory test.

Test	Result	Reference range	Units
Erythrocyte sedimentation rate (ESR)	7	0-15	mm/hr
C-reactive protein (CRP)	0.2	<1.0	mg/L
Rheumatoid factor (RF)	8	<14	IU/mL
Anti-cyclic citrullinated peptide (anti-CCP) antibodies	12	<20	U/mL

Foot radiographs documented severe osteolysis affecting both the proximal interphalangeal (PIP) and distal interphalangeal (DIP) joints of the 2nd, 3rd, 4th, and 5th toes of the left foot, with more than 80% bone loss evident in the affected phalanges. The radiographic changes demonstrated the characteristic "pencil-in-cup" destructive pattern, with extensive bone resorption creating sharp, pencil-like proximal bone ends articulating with widened, cup-shaped distal bone surfaces. Articular subluxation was present in all affected interphalangeal joints, with radiographic confirmation of the clinically observed telescoping deformities showing sequential bone collapse and digital shortening.

**Figure 5:**
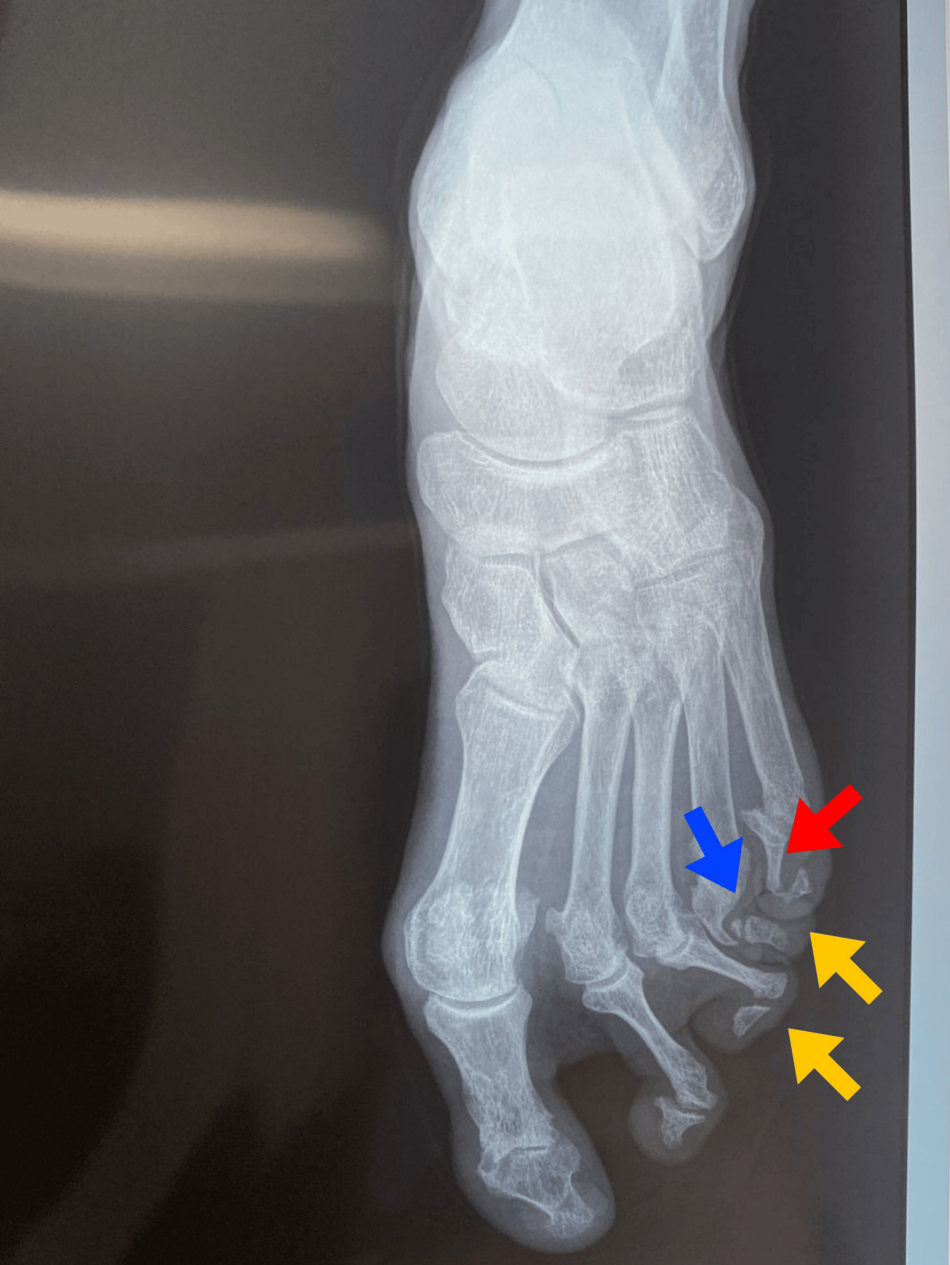
Radiograph showing osteolysis (yellow arrows), pencil-in-cup deformities (red arrow), and telescoping digits (blue arrow) in 2nd–5th toes. Note the sparing of thumb joints.

Radiological assessment using the Sharp-van der Heijde modified scoring method for psoriatic arthritis revealed a score of 7-8 points, classified as moderate joint damage (Figure 5).

The Sharp-van der Heijde modified scoring system for psoriatic arthritis classifies joint damage into three categories based on radiological findings. Mild damage (0-3 points) represents minimal structural changes, with discrete erosions or initial joint space narrowing, indicating early joint damage with a good prognosis when adequate treatment is provided. Moderate damage (4-10 points) demonstrates evident structural changes, characterized by small to moderate erosions and/or significant joint space narrowing, reflecting disease progression that requires therapeutic optimization. Severe damage (>10 points) indicates extensive joint destruction, with severe erosions, ankylosis, or joint fusion, representing irreversible damage associated with significant functional limitation.

These findings indicate the presence of evident structural changes with bone erosions and joint space narrowing, suggesting active disease progression. The obtained score reflects significant joint deterioration that warrants consideration for intensification of disease-modifying treatment to prevent further structural destruction and preserve joint function [5,7].

The diagnosis of psoriatic arthritis was established using the updated CASPAR criteria [8], achieving four points: current cutaneous psoriasis (two points), documented history of psoriasis (one point), and negative rheumatoid factor (one point). The classification of arthritis mutilans was based on major radiographic criteria, including severe osteolysis with greater than 80% bone resorption, clinically and radiographically documented telescopic deformity, and a "pencil-in-cup" pattern confirmed by radiographs.

Clinical criteria fulfilled included severe articular deformity, loss of articular function, visible digital shortening, and marked articular instability. The severity criteria were satisfied by the involvement of multiple joints with osteolysis and significant functional disability, as evidenced by a HAQ-DI of 2.8, requiring assistance for dressing and ambulation with a cane. The case evolution demonstrates a critical inverse correlation between therapeutic adherence and disease progression, with identifiable determinant tipping points influencing the final clinical outcome.

## Discussion

AM represents the most severe phenotype of psoriatic arthritis. It is characterized by aggressive osteolysis leading to the pathognomonic "telescoping digits" [7]. The condition has a low population prevalence of 3.69/1,000,000 with male predominance (M:F = 1.9:1) [7]. Our 61-year-old male patient fits this demographic profile. However, he exhibits three clinically significant distinguishing features.

The involvement of the 2nd-5th toes without hallux pathology is notable. This pattern contrasts markedly with the classical distal interphalangeal joint (DIPJ) and thumb predominance consistently reported in the literature [8,15,16]. This toe-predominant pattern suggests the existence of a clinically distinct subset of AM. Such cases warrant specialized clinical surveillance [17,18]. This anatomical distribution pattern challenges the conventional understanding of AM presentation. It may require revised classification criteria.

The absence of antecedent dactylitis is particularly noteworthy, as dactylitis is documented in 29-64% of established AM cases according to longitudinal cohort studies [12,17]. This finding may suggest that osteolysis can develop through alternative inflammatory pathways. The process could potentially occur via direct synovial cytokine release, thereby bypassing the typical entheseal inflammation cascade. However, histopathological and immunological evidence would be required to confirm such novel pathways [12].

The presence of unequivocal osteolysis on conventional radiographs obviates the need for advanced imaging modalities [15]. The literature emphasizes the value of high-resolution peripheral quantitative computed tomography (HR-pQCT)/MRI for early disease detection [15]. However, this case demonstrates that conventional radiography maintains its definitive character in advanced AM. Advanced modalities should be reserved for ambiguous presentations [15,17]. This approach optimizes diagnostic resource allocation.

Our patient with AM demonstrated normal CRP/ESR values alongside severe osteolysis. This validates the inflammation-damage dissociation described in recent literature [17]. This finding highlights a crucial clinical point. Disease activity indices based on acute-phase reactants (Disease Activity Index for Psoriatic Arthritis/Psoriatic Arthritis Disease Activity Score) prove insufficient for assessing AM. Clinical evaluation requires tools that prioritize structural damage assessment over inflammatory markers [17].

The rapid progression observed in this case is significant. Severe damage developed within 10 years of onset. This correlates with established predictors, including high joint burden, smoking history, and elevated BMI [19]. The quantified risk assessment reveals important findings. The combination of smoking (15 pack-years) and overweight BMI (28.3 kg/m²) generated a synergistic 4.1-fold increased risk for accelerated joint destruction [19]. These modifiable risk factors represent critical intervention targets.

These observations have several important implications. First, they validate phenotypic heterogeneity in destructive psoriatic arthritis. This supports the concept of distinct AM subsets [17]. Second, they refine imaging algorithms based on disease stage. This emphasizes conventional radiography for advanced cases [15,17]. Third, they highlight toe-predominant AM as a clinically distinct subset requiring specialized monitoring [17,19]. Finally, they demonstrate the critical importance of early intervention and therapeutic adherence in preventing irreversible joint destruction [16-18].

The severe functional impairment documented by our patient's HAQ-DI score of 2.8 warrants particular emphasis. This is important within the context of AM outcomes research. Validation studies have established that HAQ-DI scores exceeding 2.0 in psoriatic arthritis patients correlate with irreversible disability. These scores also correlate with significantly reduced quality of life measures [16]. Longitudinal studies demonstrate additional concerning findings. Patients with destructive arthritis phenotypes achieving HAQ-DI scores above 2.5 rarely show meaningful functional improvement. This occurs despite aggressive therapeutic intervention [17,18]. This functional assessment data reinforces an important clinical principle. Early recognition and prevention of AM represent the primary therapeutic goal. Rehabilitation potential becomes severely limited once telescoping deformities develop.

The patient's current evaluation for biological therapy represents the appropriate next step. Although delayed, this is warranted given the failure of multiple conventional DMARDs and the presence of severe structural damage requiring aggressive immunosuppressive intervention [16-18]. This case reinforces the need for sustained therapeutic engagement. The goal is to prevent irreversible joint destruction in this devastating arthritis phenotype.

## Conclusions

This case illustrates the devastating progression of arthritis mutilans in psoriatic arthritis, underscoring the critical importance of sustained therapeutic intervention. Periods of treatment discontinuation and poor adherence were temporally correlated with accelerated joint destruction. The absence of dactylitis despite severe destruction represents an atypical presentation warranting investigation, while normal inflammatory markers contrast with persistent synovial inflammation detected through advanced imaging.

The delay in biological therapy likely contributed to irreversible joint damage, reinforcing the recommendation for early aggressive treatment in high-risk patients. Preventing arthritis mutilans requires appropriate therapeutic selection, continuous patient engagement, rigorous adherence monitoring, and constant surveillance with advanced imaging, particularly in those with a history of treatment interruptions.
